# *Staphylococcus aureus* in Intensive Pig Production in South Africa: Antibiotic Resistance, Virulence Determinants, and Clonality

**DOI:** 10.3390/pathogens10030317

**Published:** 2021-03-08

**Authors:** Ncomeka Sineke, Jonathan Asante, Daniel Gyamfi Amoako, Akebe Luther King Abia, Keith Perrett, Linda A. Bester, Sabiha Y. Essack

**Affiliations:** 1 Antimicrobial Research Unit, College of Health Sciences, University of KwaZulu-Natal, Durban 4000, South Africa; nsineje@gmail.com (N.S.); josante33@yahoo.com (J.A.); essacks@ukzn.ac.za (S.Y.E.); 2 Biomedical Resource Unit, College of Health Sciences, University of KwaZulu-Natal, Durban 4000, South Africa; besterl@ukzn.ac.za; 3 Centre for Respiratory Diseases and Meningitis, National Institute for Communicable Diseases, Johannesburg 2131, South Africa; 4 Epidemiology Section, KwaZulu-Natal Agriculture & Rural Development-Veterinary Service, Pietermaritzburg 3201, South Africa; keith.perrett@kzndard.gov.za

**Keywords:** *Staphylococcus aureus*, antibiotic resistance, foodborne pathogens, multidrug resistance, MRSA, pig production chain, South Africa, genetic diversity, virulence determinants, molecular epidemiology

## Abstract

Although *Staphylococcus aureus* is a major threat to the veterinary, agricultural, and public health sectors because of its zoonotic potential, studies on its molecular characterisation in intensive animal production are rare. We phenotypically and genotypically characterised antibiotic-resistant *S. aureus* in intensive pig production in South Africa, using the farm-to-fork approach. Samples (*n* = 461) were collected from the farm, transport vehicles, and the abattoir using the World Health Organisation on Integrated Surveillance of Antimicrobial Resistance (WHO-AGISAR) sampling protocol. Bacteria were isolated using selective media and identified using biochemical tests and polymerase chain reaction (PCR). Phenotypic resistance was determined using the disk diffusion method. Selected resistance and virulence genes were investigated using PCR. Clonality among the isolates was determined using the repetitive element sequence-PCR. In all, 333 presumptive staphylococcal isolates were obtained, with 141/333 (42.3%) identified as staphylococci biochemically. Ninety-seven (97; 68.8%) were confirmed as *S. aureus* using PCR, 52.6% of which were identified as methicillin-resistant *S. aureus* (MRSA) through the *mec*A gene. All the 97 *S. aureus* isolates (100%) were resistant to at least one of the antibiotics tested, with the highest resistance observed against erythromycin and clindamycin (84.50% each), and the lowest observed against amikacin (2.10%); 82.47% (80/97) were multidrug-resistant with an average multiple antibiotic resistance index of 0.50. Most of the phenotypically resistant isolates carried at least one of the corresponding resistance genes tested, *erm*C being the most detected. *hla* was the most detected virulence gene (38.14%) and *etb* was the least (1.03%). Genetic fingerprinting revealed diverse MRSA isolates along the farm-to-fork continuum, the major REP types consisting of isolates from different sources suggesting a potential transmission along the continuum. Resistance to antibiotics used as growth promoters was evidenced by the high prevalence of MDR isolates with elevated multiple antibiotic resistance indices >0.2, specifically at the farm, indicating exposure to high antibiotic use environments, necessitating antibiotic stewardship and proper infection control measures in pig husbandry and intensive pig production.

## 1. Introduction

According to recent World Health Organisation estimates, food contamination affects over 600 million people worldwide, with over 420,000 dying every year [1]. This condition is further exacerbated by the presence of antibiotic-resistant bacteria in contaminated foods. Today, antibiotic resistance is a global public health concern [2,3] that poses a severe threat to human and animal health [4,5]. The escalating antibiotic resistance rate may be attributed to the excessive and inappropriate antibiotics use in humans and animals, including in animal husbandry [2,4].

*Staphylococcus aureus* is a bacterium that exists as either a commensal or pathogen in humans and animals [6]. Its success as a pathogen may be attributed to the production of many virulence factors, including enterotoxins, leucocidins, exfoliative toxins, haemolysins, and immune-modulatory factors [7,8,9] that promote colonisation, tissue damage, and infection while facilitating the evasion of host defence mechanisms. Moreover, its ability to resist a wide range of antibiotics has led to limited therapeutic options for treating its infections [10]. *S. aureus* has shown resistance to most β-lactam antibiotics, linezolid, daptomycin, and vancomycin, which are the last-resort antibiotics for Gram-positive bacteria [11]. Its resistance mechanisms encompass the enzymatic inactivation of antibiotics, alteration of the target penicillin-binding proteins that decrease the antibiotic’s binding affinity, and efflux pumps that remove antibiotics from the bacteria’s cytoplasm. Resistance is acquired through mutations and horizontal gene transfer of resistance genes on various mobile genetic elements such as plasmids, bacteriophages, and transposons [10,12]. Methicillin resistance in *S. aureus* is mediated by the *mec*A gene that is harboured by a mobile genetic element, the staphylococcal cassette chromosome mec (*SCCmec*) [13].

Epidemiologically, methicillin-resistant *S. aureus* (MRSA) is divided into three classes, hospital-acquired MRSA (HA-MRSA), community-associated MRSA (CA-MRSA), and livestock-associated MRSA (LA-MRSA) [14]. Pigs were identified as important reservoirs for LA-MRSA as early as 2004, and LA-MRSA lineages have been currently reported in humans, suggesting a possible transmission from animals to humans, blurring the epidemiology of MRSA [15].

Antibiotic overuse is the primary driving force of resistance in pig production. Antibiotics are used as growth promoters for metaphylaxis and prophylaxis to improve health, produce high-quality products, and increase overall production yield [16]. In 2016, it was estimated that South Africa consumes 200,000 tons of pork, which is the second most consumed source of meat after chicken [17]. Due to high demand, different antibiotics are extensively used during food animal production [16,18]. The high demand for pork also imposes the need to adopt intensive production approaches, requiring the farming of many animals within limited and confined spaces. However, this approach has the downside of promoting stress and increasing disease transmission within the animal farm [19], thus requiring extensive antibiotics use to treat sick animals. The use of these antibiotics, including the critically important and clinically relevant ones in food animals, can create a selective environment for the emergence of multidrug-resistant pathogenic strains.

Despite the safety issues associated with drug-resistant bacterial contaminants in food, there are limited studies on antibiotic-resistant *S. aureus* in intensive pig production or the possibility of transmission to humans in South Africa. Furthermore, no study has investigated this along the pig farm-to-fork continuum in Africa. Therefore, we elucidated the molecular epidemiology of antibiotic-resistant *S. aureus* in an intensive pig production chain in uMgungundlovu District KwaZulu-Natal, South Africa, using the farm-to-fork approach. This study would provide the foundations for implementing measures to curb antibiotic use in food animals and identify areas along the continuum that may be prioritised in such interventions.

## 2. Results

### 2.1. Staphylococcus Aureus Detection Rate in the Pig Production Chain

A total of 333 presumptive staphylococcal isolates were obtained throughout the pig production chain based on culture characteristics. However, the biochemical analysis yielded 141 (42.3%) *Staphylococcus* isolates, of which 97 (68.8%) were confirmed as *S. aureus* through PCR. The least number of *S. aureus* isolates was obtained on Week 4 from litter and faecal samples. Most *S. aureus* isolates were obtained on Week 7, while no isolates were obtained on Week 9. In addition, no isolates were recovered from caecal samples (Table 1).

Furthermore, 51 (52.6%) of the 97 *S. aureus* isolates were positive for the *mec*A gene, confirming them as MSRA.

### 2.2. Antibiotic Susceptibility Profiles

**Figure 1:**
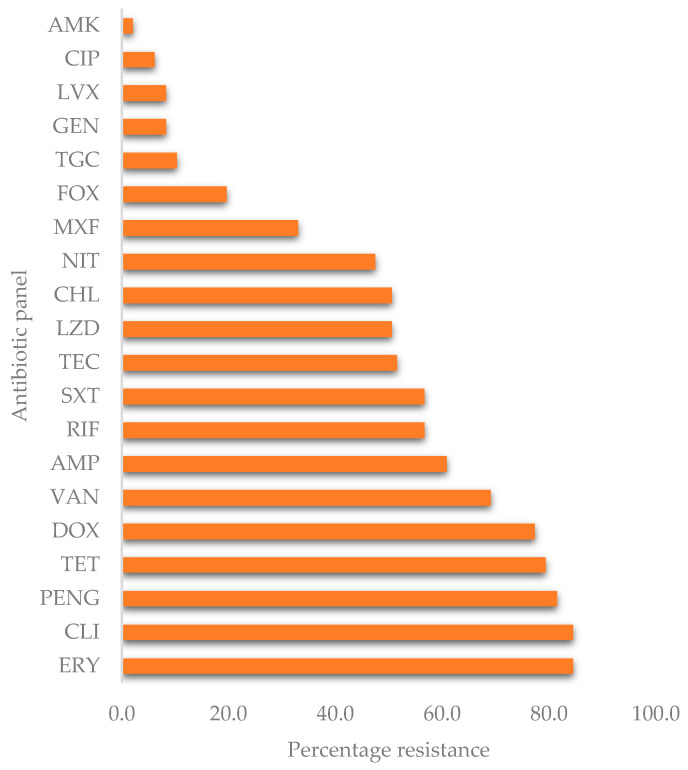
Overall percentage resistance of *S. aureus*. AMP = ampicillin, PENG = penicillin-G, CIP = ciprofloxacin, MXF = moxifloxacin, LVX = levofloxacin, LZD = linezolid, FOX = cefoxitin, AMK = amikacin, GEN = gentamicin, TGC = tigecycline, TET = tetracycline, DOX = doxycycline, ERY = erythromycin, CLI = clindamycin, RIF = rifampicin, SXT = sulfamethoxazole-trimethoprim, NIT = nitrofurantoin, CHL = chloramphenicol, TEC = teicoplanin, VAN = vancomycin.

The antimicrobial susceptibility was only performed on PCR-confirmed *S. aureus* isolates. These isolates displayed varying percentages of resistance to the various antibiotics tested (Figure 1). The highest resistance was observed against clindamycin (84.50%) and erythromycin (84.50%), while the lowest resistance was against amikacin (2.10%). Overall, all the isolates (100%) were resistant to at least one of the 20 antibiotics tested. There was substantial resistance to the glycopeptide antibiotics, vancomycin (69.10%), and teicoplanin (51.50%).

**Figure 2:**
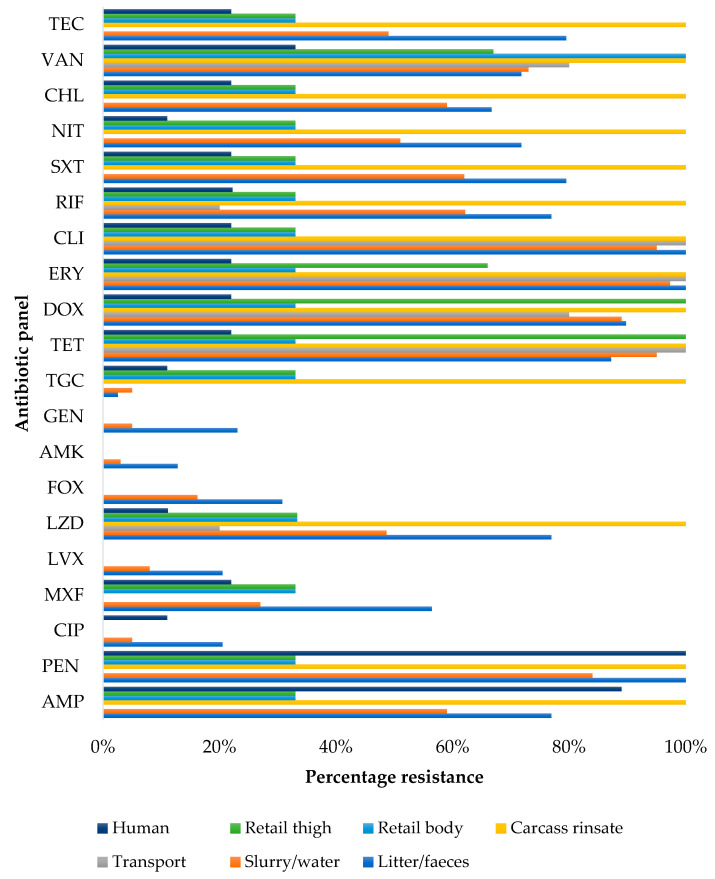
Percentage resistance of *S. aureus* isolates stratified by source. AMP = ampicillin, PENG = penicillin-G, CIP = ciprofloxacin, MXF = moxifloxacin, LVX = levofloxacin, LZD = linezolid, FOX = cefoxitin, AMK = amikacin, GEN = gentamicin, TGC = tigecycline, TET = tetracycline, DOX = doxycycline, ERY = erythromycin, CLI = clindamycin, RIF = rifampicin, SXT = sulfamethoxazole-trimethoprim, NIT = nitrofurantoin, CHL = chloramphenicol, TEC: teicoplanin, VAN = vancomycin.

Furthermore, when stratified by sampling source, isolates obtained from transport samples were 100% resistant to erythromycin, clindamycin, and tetracycline, while faecal samples showed 100% resistance to penicillin-G (Figure 2).

### 2.3. Multidrug Resistance and Risk Assessment Parameters

**Figure 3:**
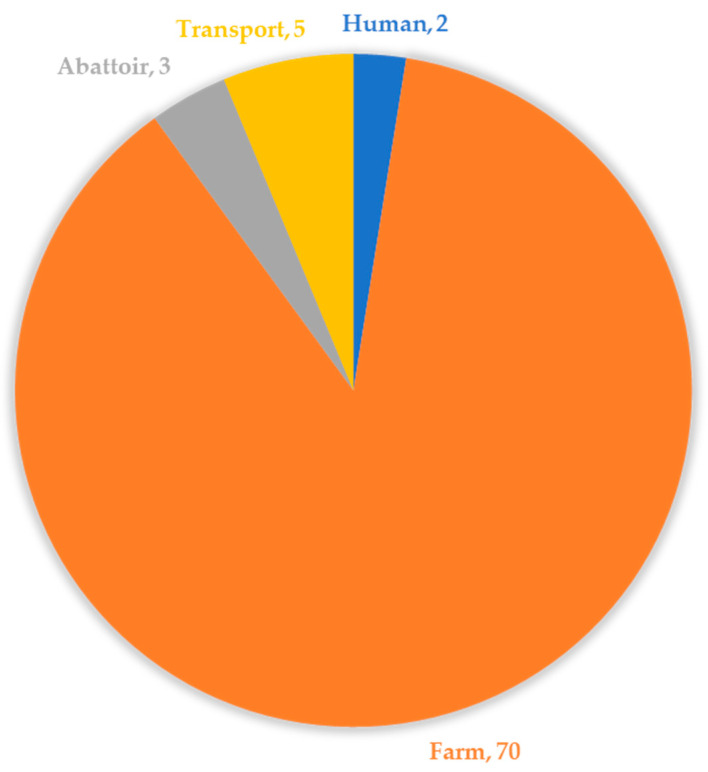
The overall distribution of multidrug-resistant *S. aureus* isolates along the pig production chain.

Out of 97 *S. aureus* isolates, multidrug resistance (MDR) was recorded in 82.47% (80/97) of the isolates, which were mostly from the farm (faeces, slurry, and litter) and the least being from humans (Figure 3).

The isolates displayed varying resistance patterns that were grouped into 56 different antibiograms, LZD-RIF-ERY-CLI-AMP-PEN-SXT-MXF-TET-DOX-NIT-CHL-VAN-TEC being the most common pattern (Table S1). Most MDR isolates (66; 82.50%) were resistant to six or more tested antibiotics (Table S1). No isolate was pan-drug resistant (i.e., showing resistance to all antibiotics tested in this study).

The overall MARI for all the isolates in the current study ranged between 0.02 and 0.95. However, most isolates recorded an MAR (multiple antibiotic resistance) index of 0.80 throughout the production chain. The highest MARI (0.95) was recorded on the farm; this isolate was resistant to 19 of the 20 antibiotics tested (Table S1). On the farm, the MARI ranged between 0.20 and 0.95 (mean = 0.50). The transport system ranged between 0.20 and 0.30 (mean = 0.25), while at the abattoir, isolates recorded MARIs of 0.75 and 0.80. The two MDR human isolates had MARIs of 0.50 (hands) and 0.85 (nasal).

### 2.4. Detection of Antibiotic Resistance and Virulence Genes

The tested resistance genes were detected at varying percentages in the isolates that showed phenotypic resistance to the corresponding antibiotics or antibiotic classes (Table 2). For example, the *erm*C gene was detected in 97.56% (80/82) of the isolates that were phenotypically resistant to erythromycin. The vancomycin resistance genes, *van*A and *van*B, were not detected, although phenotypic resistance was observed. There was no correlation between the antimicrobial resistance genes (ARGs) from the different sampling points and sources.

The distribution of virulence genes was *hla* (39%), *hld* (23%), *seb* (3%), *sed* (2%), *etb* (1%), *LukS/F-PV* (30%), and *tst* (11%). The most common virulence factor was the α-hemolysin cytotoxin encoded by the *hla* gene (Table 2). Low prevalence was recorded for exfoliating toxins encoded by *etb* and staphylococcal enterotoxin genes *seb* and *sed*. Virulence genes *eta* and sea were not detected.

### 2.5. Repetitive Element Palindromic PCR (REP-PCR)

**Figure 4:**
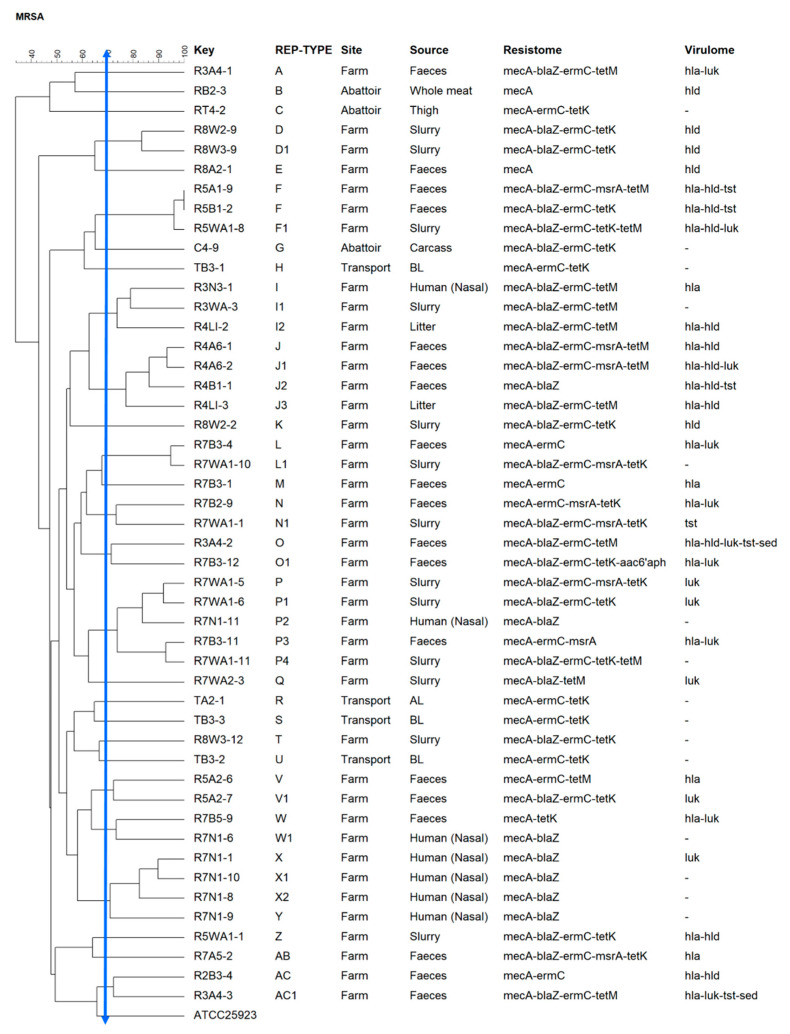
Dendrogram showing repetitive element palindromic (REP)-type groups of *S. aureus* isolates, based on the 70% similarity index, recovered along the farm-to-fork continuum. The solid blue line indicates the REP-type cut-off.

Twenty-eight (28) clusters were identified from A-AC (Figure 4). Amongst these, 38% constituted five major rep types, namely P (*n* = 5), J (*n* = 4), F (*n* = 3), I (*n* = 3), and X (*n* = 3). The largest clonal cluster was P (*n* = 5), with isolates originating from the farm (faeces, slurry, and human samples). J (*n* = 4) contained Week 4 isolates from faeces and litter. Repetitive element palindromic (REP)-F (*n* = 3) consisted of Week 5 faecal and slurry isolates. REP-type X (*n* = 3) consisted of Week 7 isolates obtained from human swabs, and REP-type I (*n* = 3) contained Week 3 human and slurry isolates and a Week 4 litter isolate. REP-I was the only major REP-type with isolates originating from different but consecutive sampling weeks.

## 3. Discussion

### 3.1. Prevalence of S. aureus in the Pig Production System

Many different bacteria have been implicated in foodborne disease outbreaks around the world. In the current study, staphylococcal species were isolated from different sources throughout the production chain. Overall, 69% (*n* = 97) of the total number of isolates were identified as *S. aureus*, of which 52.6% were confirmed as MRSA using molecular techniques. This prevalence was considerably higher than the 12% reported in another South African study conducted in 2017 assessing MRSA prevalence in commercial pig herds in the Western Cape, KwaZulu Natal, and Gauteng [20]. A lower prevalence was also reported in another South Africa study assessing the formal (30%) and informal (50%) meat sectors with isolates obtained from meats samples in abattoirs and slaughtering points [21]. These differences in prevalence may reflect the sampling framework used. Unlike the current study, most studies have been performed at single points along the continuum [22]. The differences in percentages could also be due to the methods used. Unlike our study that used PCR to detect the mecA gene, the previous studies only relied on culture using selective media and biochemical tests to confirm MRSA in their isolates. While being valuable in their respects, such narrow sampling could give an incomplete picture of the epidemiology of *S. aureus* in the food production chain. Thus, the farm-to-fork sampling approach used in the current study, as recommended by the WHO, provides a better understanding of the microbial pathogens’ distribution in intensive production systems. This could also highlight the potential transmission along the continuum and identify hotspots needing prompt attention. The MRSA prevalence rate may also differ according to geographic location and herd size [23]. Globally, high prevalence rates (>50%) have been reported in the USA, Germany, Italy, and Sri Lanka [6,24,25,26]. Nevertheless, the scarcity of information in South Africa, as in many developing countries, on the current situation or possible dissemination of these bacterial pathogens through the food production chain remains a concern [27].

### 3.2. Antimicrobial Resistance Profile of S. aureus Isolated from the Pig Production Chain

Pig production is one of the leading sources of meat protein in South Africa, after poultry. However, the intensive conditions under which pigs are housed during production are a risk factor for spreading disease, resulting in high antibiotic use to control and treat infections [28]. The routine use of antibiotics as growth promoters for prophylaxis, metaphylaxis, and treatment exerts selective pressure for developing and escalating antibiotic resistance [26]. This creates large reservoirs of antibiotic-resistant bacteria, including MRSA, colonising the nares, skin, and rectum of the pigs and occupationally exposed workers. For example, significantly high resistance percentages, including high MDR rates, have been reported to some antibiotics frequently used in veterinary medicine for animal husbandry, such as tetracycline, penicillin, erythromycin, and sulphonamides [29]. The transmission of resistant bacteria between animals and humans has also been reported in many studies [30,31,32]. For example, a report on antimicrobial use and resistance in Africa indicated a 100% prevalence of MDR *E. coli* in South Africa with isolates highly resistant to sulphonamides, tetracycline, and penicillin [33]. The high percentage of resistance to these antibiotics may be because they are widely used, favoured by their low cost and availability [32]. In veterinary medicine, penicillins are commonly used for prophylaxis and treatment of urinary tract infection and have been frequently detected in foodborne *S. aureus* [34,35].

According to a document published by the WHO on critically important antibiotics for human medicine in 2018 [36], some classes of antibiotics used in food animals are also used to treat human infections. Hence, their indiscriminate use in animal production may cause resistance, compromising their efficacy in human infections [32]. Although the European Union has banned some of these antibiotics as growth promoters, they are still used in South Africa [29,37]. The >50% resistance observed for erythromycin, clindamycin, penicillin-G, tetracycline, and doxycycline may be attributed to the use of these antibiotics to promote growth, prevent, and treat infections. Similar resistance patterns reported in China and Portugal in pig production were correlated with overuse [38,39].

The resistance profiles observed in the current study suggest cross-resistance to frequently used antibiotic analogues for growth promotion. For example, resistance to erythromycin and clindamycin may be attributed to the use of tylosin and kitasamycin in the feed [22,40]. In addition, tetracycline and doxycycline analogues are commonly used for the treatment of respiratory infections. These antibiotics may be administered through drinking water or feed over a prolonged period [32]. The Stock Remedies Act No.36 of 1947 has made antibiotics available over the counter for growth promotion and prophylaxis. This laxity could increase antibiotic resistance in foodborne pathogens in the food production chain in South Africa. However, as recommended by the WHO, a ban on such use is already effective in the European Union for the last decade [41]

Furthermore, the current study revealed that over 82% of the isolates were MDR, with 56 antibiograms, indicating diverse resistance patterns. This observation intimates the mobilisation and easy exchange of antibiotic resistance genes between isolates across the farm-to-fork continuum. The diversity of resistance patterns and the high MDR rate highlights the need for antibiotic stewardship to ensure prudent antibiotic use for animal production, as it may have grave consequences for human and environmental health [42]. The overall large number of isolates with MARI > 0.2 (average = 0.47) further illustrates the selection pressure of excessive antibiotic use, indicating that these isolates were from environments of high antibiotic exposure, as would be expected if antibiotics are used for growth promotion, metaphylaxis, or prophylaxis [43,44]. Comparatively, an average MAR index > 0.3 was reported in India in pork [45].

The possible dissemination of MDR strains along the production chain due to the handling and contamination emphasises the need to monitor and enforce infection prevention and control measures at each stage in the food production chain. For example, the two MDR human isolates had MARIs of 0.5 (hands) and 0.85 (nasal). While the number of isolates was small, the high MARI values indicate a potential health hazard for the farmworkers. However, it cannot be concluded that the isolates identified in the humans originated from the farm, as human samples were not collected before the workers entered the farm. Similarly, isolates in the abattoir recorded MARI values between 0.75 and 0.80. Although these isolates likely came from the farm, it may not be concluded that they were from the same batch of pigs, since the abattoir serves many other farms within the district. This could be further supported because *S. aureus* was also isolated from the truck before our animals were loaded. These observations indicate that the transmission of microorganisms along the farm-to-fork continuum, especially antibiotic-resistant ones, exhibits a complex dynamic that requires further investigation using advanced molecular tools such as whole-genome sequencing.

### 3.3. Antibiotic Resistance Mechanisms

Although there was a general agreement between phenotypic and genotypic resistance, there were a few discrepancies. The most common resistance gene detected in this study was the *erm*C gene in over 90% of the isolates resistant to erythromycin, while another macrolide resistance gene, *msr*A, was detected at much lower levels (Table 2). The *erm*C gene facilitates the methylation of the 23S rRNA ribosome’s active site, triggering conformational changes, resulting in drug binding inhibition [7,46], while *msrA* encodes for an ATP-dependent efflux pump. Resistance to erythromycin reportedly co-selects resistance to other antibiotics such as the type B streptogramin (MLS_B_) and lincosamides. The frequent use of antibiotics such as streptogramin, virginiamycin, or tylosin to promote growth in farm animals through feeds has accounted for increasingly high numbers of isolates carrying the macrolide resistance genes [10].

The use of virginiamycin (streptogramin associated with resistance to quinupristin-dalfopristin), amongst others, for growth enhancement, was endorsed by the Pig Veterinary Society of the South African Veterinary Association in its policy document on “guidelines for the use of antimicrobials in the South African pig industry” [47]. Additionally, a survey by Eager et al. on the animal use of antimicrobials in South Africa reported high tylosin sales as a registered growth promoter [29]. This raises concerns because tylosin was banned alongside virginiamycin, spiramycin, and bacitracin in the EU based on WHO recommendations due to their chemical and structural homologies to antibiotics used in humans [48].

The prevalence of the *bla*Z gene was reported in 88.75% of penicillin-resistant isolates. In *S. aureus*, this gene is found on transposon Tn522 located in plasmid pI524. *blaZ* produces β-lactamase, which inactivates penicillin by hydrolysing its β-lactam ring [10]. Zehra et al. earlier found the *bla*Z as the most prevalent resistance gene in *S. aureus* in bovine and swine from Punjab, India [49]. Furthermore, tetracycline resistance is conferred by two mechanisms: the active efflux of drugs, facilitated by *tet*K and *tet*L, and ribosomal protection due to the acquisition of *tet*M and *tet*O [50]. Our study’s isolates displayed a higher prevalence of *tet*K than *tet*M (Table 2), accounting for the tetracycline resistance observed in the phenotypically resistant isolates. This finding was similar to that of Sieber et al. in a Danish study on LA-MRSA in pigs and humans [51]. However, other studies have reported a comparatively higher prevalence of *tet*M than *tet*K [52,53]. It has been established that most MRSA harbour both *tet*K and *tet*M, which confer resistance to all tetracycline antibiotics [54].

Most isolates that were phenotypically resistant to gentamicin harboured the *aac(6′)-aph(2”*) gene. However, the absence of this in two isolates could suggest that other aminoglycoside resistance mechanisms that were not investigated in this study might have conferred resistance. Nevertheless, the low prevalence of resistance (phenotypic and genotypic) may imply that aminoglycosides can still be used to treat clinical staphylococcal infections successfully; hence, its prudent use is advised in food animal production.

It has been reported that using avoparcin to promote growth in agriculture has facilitated the emergence of glycopeptide-resistant enterococci [55]. The resistance genes involved have been disseminated into other Gram-positive bacteria, including MRSA. The emergence of vancomycin-resistant MRSA is a cause for concern, considering that vancomycin is a drug of choice for resistant hospital-acquired infections [48]. Although the current study reported phenotypic resistance to vancomycin, the targeted *van*A and *van*B genes were not detected. This was in line with another South African study on *Staphylococcus* in farm animals, which revealed that 12% of the phenotypically vancomycin-resistant MRSA did not harbour the *van*A and *van*B resistance genes [56]. This could be attributable to other plasmid-mediated vancomycin genes that were not investigated in the current study, such as *van*C, *van*D, *van*E, *van*F, and *van*G [57]. More so, vancomycin resistance may also be caused by decreased permeability by thickening the cell wall, thus inhibiting/decreasing vancomycin availability to intracellular target molecules [58]. High percentage resistance to teicoplanin was also observed in the study; however, this was not peculiar, as cross-resistance between glycopeptides has been reported [52,53].

Further studies involving whole-genome sequencing (WGS) to detect unknown or novel mechanisms would be useful to delineate the genetic basis of resistance [14]. Lastly, isolates showed over 50% *mec*A gene prevalence, which is not surprising, as the MDR rate was also high. Isolates phenotypically resistant to cefoxitin but lacking the *mecA* gene could be due to alternative mechanisms of cefoxitin resistance such as *mec*C [54].

### 3.4. Virulence Determinants

*S. aureus* harbours various virulence determinants that contribute to its pathogenicity. Therefore, food animals may be a source of transmission of pathogenic strains in production facilities to humans and the environment [58]. Isolates predominantly harboured the *hla* gene (Table 2), an α-haemolysin cytotoxin, which contributes to biofilm formation in epithelial tissues, promoting infections and slowing down wound healing [59]. Staphylococcal enterotoxins (*sea, seb*, and *sed*) were recovered at lower rates, which agreed with other animal studies [60,61]. For example, Dweba et al. reported a prevalence of 6.4% (*sea*) and 6% (*sea*) for the gene amongst different animal species in South Africa [61]. Staphylococcal enterotoxins are usually associated with food poisoning, with *seb* considered a potential inhaled bioweapon [62,63]. Moreover, *S. aureus* may produce Panton-Valentine leucocidin (PVL), a pore-forming toxin encoded by phage-encoded genes [64]. PVL is also considered a genetic marker for CA-MRSA due to its prominence in this epidemiological class [64]. A substantial number of isolates carried the *PVL* gene, corroborating a study conducted on *S. aureus* isolated from backyard-raised pigs and pig workers in Nigeria, with 27% of isolates harbouring *PVL* [65]. However, although *PVL* has been associated with necrotising pneumonia and joint infection in humans, its role in pigs is not thoroughly investigated; hence, the current findings should be interpreted with caution [66], as its presence does not necessarily imply diseased animals.

### 3.5. Clonal Relatedness of Isolates

Vancomysin has been regarded as the drug of choice to treat infections caused by MRSA; however, the increased resistance of these bacteria to vancomycin warrants rapid typing methods to characterise MRSA, as they have also been isolated from meat and meat products [67]. Although pulse-field gel electrophoresis (PFGE) has been regarded as the gold standard for typing MRSA, REP-PCR is more practical, time-efficient, and cost effective than other typing methods [68]. In addition, REP-PCR yields comparative results to PFGE [69] while outperforming more recent methods such as multilocus sequence typing (MLST) and PFGE in some instances [70].

Thus, using REP-PCR in the current study, the 48 MRSA isolates selected for typing yielded 28 REP types (A-AC) based on a 70% similarity index, with the majority concentrated within the farm environment. Five major REP types were identified on the farm, with isolates sourced from faeces, slurry, human swabs, and litter samples, usually at the same time points. Clonal relatedness was evident in isolates from pig faeces, human swabs, and the environment (slurry/litter), belonging to six REP types (F, I, J, L, N, and P). Although this was not surprising, due to the proximity between these sample sources, it further strengthens the knowledge of potential transmission of microbial species between humans, animals, and the environment within animal farms. Some isolates belonging to the same clones carried some similar resistance and virulence genes, although there was extensive diversity in the resistance, virulence, and clonal profiles. Such a high diversity could also be due to the small number of isolates included in the experiment. Selecting a few isolates from each sampling point may introduce bias that could allocate phenotypically similar isolates to different clonal groups. However, such selection could not be avoided, as the number of isolates obtained depended on the number of positive samples. Therefore, studies involving a larger number of isolates could provide a better picture of the clonality along the farm-to-fork continuum. A similar trend was observed in a study by Neyaz et al. on the characterisation of *S. aureus* from various meat products where a high prevalence of tetracycline resistance was reported in two different clones [65]. A 2019 study in Italy using other typing methods, including WGS, reported 94.1% of human MRSA isolates belonging to the same epidemiological group as swine MRSA isolates [26]. Of note, although REP-PCR has a shorter turnaround time, it is less discriminatory; therefore, further studies involving more resolute typing approaches such as WGS are recommended [71]. Nevertheless, it should also be noted that the diversity reported in the current study was based on a 70% similarity cut-off value and that changing this index could alter the number of REP-types in any given experiment.

## 4. Materials and Methods

### 4.1. Study Site and Sample Collection

The study was conducted in the uMgungundlovu District Municipality in KwaZulu-Natal (KZN), South Africa. This district is one of the largest districts in the KZN Province and contains all the major intensive food animal farms in the region.

Four hundred sixty-one (461) samples were collected from Farm P, its occupationally exposed farmworkers, farm environments, and associated abattoir over 18 weeks (September 2018–January 2019). The samples were collected across the farm-to-fork continuum (animal faeces on the farm, transport, and post-slaughter) as per the World Health Organisation on Integrated Surveillance of Antimicrobial Resistance (WHO-AGISAR) protocol [72]. The farm, transport, and abattoir samples were collected as previously described [73]. Block sampling was used to make sure the entire herd was well represented. Faecal and slurry samples were collected bi-weekly from day 0 to 126 (slaughter).

Additionally, hand and nasal swabs were obtained from farm employees. On the 18th week, the same herd was followed to the abattoir. Samples were collected from the transport (truck, before and after loading the pigs) and at different stages from slaughter to packaging for human consumption from the same herd sampled on the farm [73]. All collected samples were immediately stored in a cooler box containing ice packs and transported to the laboratory for processing within 4 h of sampling.

### 4.2. Isolation and Identification of Staphylococcus aureus

#### 4.2.1. Isolation of *S. aureus*

The samples were inoculated into tryptone soya broth (TSB) (Basingstoke, Hampshire, England) and incubated at 37 °C for two hours while shaking (100 rpm). Then, these samples were streaked on HiCrome Aureus Agar Base (Himedia Laboratories, Mumbai, India) and incubated overnight at 37 °C under aerobic conditions. After incubation, colonies showing a unique brown-black colour with a clear zone were streaked onto mannitol salt agar (Himedia Laboratories, Mumbai, India) for further screening. Presumptive *S. aureus* colonies were examined for coagulase-activity by the tube plasma test and DNAse tests [74]. The presumptive *S. aureus* colonies were maintained at −80 °C in 10% glycerol stocks for further analysis.

#### 4.2.2. Molecular Confirmation of *Staphylococcus aureus* and Identification of Methicillin-Resistant *S. aureus* (MRSA)

DNA was extracted using the GeneJet Genomic DNA purification kit according to the manufacturer’s instructions (ThermoFischer Scientific, Waltham, MA, USA). The concentration and purity of the DNA were determined spectrophotometrically using the Nanodrop ND-1000 Spectrometer (ThermoFisher Scientific, Waltham, MA, USA). The extracted DNA was used as the template in the PCR. Molecular confirmation was performed using *S. aureus* species-specific primers for the *nuc*A gene, which codes for a thermostable nuclease [75]. The primer sequences used were *nuc*AF 5′-GCGATTGATGGTGATACGGTT-3′ and *nuc*AR 5′-AGCCAAGCCTTGACGAACTAAAGC-3′ (Inqaba Biotechnical Industries (Pty) Ltd., Pretoria, South Africa), generating a 270-base pair fragment [75]. PCR was performed in a 20 μL reaction volume with 3 μL DNA template, 10 μL Luna^®^ Universal qPCR master mix (Biolabs, New England Ipswich, MA, USA), 0.5 μL from each forward and reverse *nuc*A primers (20 µM), and 6 μL of nuclease-free water (Thermo Scientific, Waltham, MA, USA). The PCR protocol included activation for 5 min at 94 °C; 35 cycles of 30 s at 94 °C (denaturation), 45 s at 62 °C (annealing), and 45 s at 72 °C (elongation), and a final extension step of 10 min at 72 °C. All reactions were carried out in a T100^TM^ thermal cycler (BioRad, Hercules, CA, USA). The PCR products were subjected to electrophoresis on a 1.5% agarose gel stained with ethidium bromide in 0.5 Tris-acetate-EDTA (TAE) buffer (HiMedia, Mumbai, India) at 120V for 1 h. Gels were visualised in a Gel Doc^TM^ XR + imaging system (Bio-Rad, Hercules, CA, USA). The confirmed isolates were coded according to their collection site. Then, isolates that were positive for the *nuc*A gene were tested for the presence of the *mec*A gene to identify MRSA isolates using primers and PCR conditions described in Table S2.

*S. aureus* ATCC 25,923 and *S. epidermidis* ATCC 12,228 were used as the positive and negative controls for the presumptive phenotypic and genotypic identification experiments.

### 4.3. Antimicrobial Susceptibility Testing (AST)

The isolates’ antibiotic susceptibility profiles were determined using the disk diffusion method on Mueller–Hinton Agar (Merck (PTY) Ltd., Modderfontein, South Africa) and interpreted according to the European Committee on Antimicrobial Susceptibility Testing (EUCAST) breakpoints [76]. The Clinical and Laboratory Standards Institute (CLSI) guidelines [77] were used for antibiotic breakpoints absent from the EUCAST 2017 guidelines. Care was taken to ensure that the recommended ≈25 mL of agar was poured in each 90 mm plate, as the agar depth/volume could affect the antimicrobial susceptibility testing (AST) results. Antibiotics were selected based on the WHO-AGISAR 2017 protocol, their availability, and frequency of use in veterinary and human medicine in the country. The following 20 antibiotics were used: penicillin G (PEN 10 μg), ampicillin (AMP 10 μg), tigecycline (TGC 15 μg), nitrofurantoin (NIT 300μg), cefoxitin (FOX 30 μg) (interpreted using EUCAST breakpoints), amikacin (AMK 30 μg), gentamicin (GEN 10 μg), ciprofloxacin (CIP 5 μg), moxifloxacin (MXF 5 μg), levofloxacin (LVX 5 μg), tetracycline (TET 30 μg), doxycycline (DOX 30 μg), erythromycin (ERY 15 μg), clindamycin (CLI 2 μg), teicoplanin (TEC 30 μg), trimethoprim-sulfamethoxazole (SXT 1.25/23.75μg), chloramphenicol (CHL 30 μg), linezolid (LZD 30 μg) and rifampicin (RIF 5 μg) (interpreted using CLSI breakpoints) (Oxoid, Basingstoke, UK). The diameters of the zone of inhibition around the disks were measured to the nearest millimetre (mm) using a ruler. The minimum inhibitory concentrations (MICs) for vancomycin (VAN) were determined through the broth microdilution method using the CLSI guidelines [77]. A methicillin-sensitive strain, *S. aureus* ATCC 29213, was used as a positive control.

### 4.4. Risk Assessment Parameters of S. aureus Isolates

Multidrug resistance is defined as resistance to one or more agents in three or more distinct antibiotic classes, and it was determined from the AST results [78]. The multiple antibiotic resistance index (MARI) was calculated as (a/b), where “a” is the number of antibiotics to which the isolates were resistant, and “b” is the total number of antibiotics to which the isolate was tested [44]. Bacteria having a MARI > 0.2 originate from a high antibiotic exposure environment, while values < 0.2 show bacteria from lower antibiotic use sources. A completely resistant isolate has a MARI of 1.0.

### 4.5. Genotypic Characterisation of Isolates’ Resistance and Virulence Potentials

Resistance and virulence genes were determined by PCR using primers (Inqaba Biotechnical Industries (Pty) Ltd., Pretoria, South Africa) and PCR conditions listed in Table S2. PCR was performed in a 20 µL reaction mixture consisting of 10 µL One Taq Master Mix (x2) (Biolabs, New England Ipswich, MA, USA), 6 µL of nuclease-free water, 0.5 µL of each primer pair (final concentration of 0.5 µM), and 3 µL of template DNA. All reactions were carried out in a T100^TM^ Thermal Cycler (Bio-Rad, Hercules, USA). Each PCR assay included a positive control and a No Template Control (NTC) consisting of the PCR mix with template DNA replaced by nuclease-free water.

### 4.6. Determination of Genetic Relatedness Using Repetitive Element Palindromic PCR (REP-PCR)

The REP-PCR was only conducted on the MRSA isolates. Each PCR reaction was carried out in a 25 µL reaction mixture containing 12.5 µL of Dream Taq (Thermo Fischer Scientific, Vilnius, Lithuania), 10.5 µL of nuclease-free water, 1 µL of GTG_5_ primer, and 1 µL of template DNA. The cycling conditions were as previously reported [72]. PCR products were subjected to electrophoresis in a 1% agarose gel in 1X Tris-acetate-EDTA (TAE) buffer containing 5 µL of ethidium bromide and run at 75 V for 3 h. The gels were visualised, and the images were captured with a Gel Doc ^TM^ XR imaging system (Bio-Rad, Hercules, California, USA). A 1 Kb DNA ladder (Biolabs, New England, Hertfordshire, UK) was used as a reference molecular weight marker. The resultant electrophoretic patterns were analysed using the BioNumerics software version 6.6 (Applied Maths NV, Belgium) using the Dice coefficient. Clustering was done through the unweighted pair group with arithmetic averages (UPGMA) using 1% tolerance and 0.5% optimisation. Clusters were identified based on a similarity of ≥ 70% [50].

### 4.7. Statistical Analysis

Descriptive statistics were used to describe the prevalence *of S. aureus* isolates, phenotypic resistance profiles, and genotypic profiles from different sources. The association between MAR index, resistance, and virulence genes was determined by performing a Chi-square test using SPSS (Statistical Package for the Social Sciences) v 20 (IBM, Armonk, USA). Results were considered statistically significant at α = 0.05.

## 5. Conclusions

This study confirmed that pigs serve as important reservoirs for MDR *S. aureus*, including MRSA, with significant zoonotic implications and transmission potentials to humans through occupational exposure. The resistance to a range of antibiotics used as growth promoters, high MDR prevalence, and MARI values suggest a transmission risk between animals and humans. This poses a challenge to food safety and human and veterinary medicine, necessitating proper surveillance, stewardship, and biosecurity programmes in intensive food animal production. However, it should be noted that although clonalilty was observed among the isolates in the current study, all major REP types were found on the farm with no transmission evidence across the farm-to-fork continuum. Therefore, while being crucial for understanding the molecular epidemiology of *S. aureus* in intensive pig farming, the results of the current study should not be over-generalised. The clonality was only based on MRSA isolates, and other staphylococci and microbial pathogens carrying resistance genes could still be transmitted across the continuum.

## Figures and Tables

**Table 1:** Distribution of presumptive staphylococcal isolates obtained throughout the study.

Week	Production Stage	Source	No. Collected
Weeks 1–17	Farm (*n* = 293)	Faeces	117
		Slurry	119
		Human (Nasal)	57
Week 18	Transport (*n* = 4)	Before Loading	4
		After Loading	4
Week 18	Abattoir (*n* = 32)	Carcass Rinsate	12
		Caecal contents	0
		Retail Meat (Whole Carcass)	4
		Retail Meat (Head)	8
		Retail Meat (Thigh)	8
	Total		333

**Table 2:** Prevalence of antibiotic resistance and virulence genes in *S. aureus* isolates.

Resistance Gene *	Prevalence	Virulence Genes **	Prevalence
*tet*M	27 (35.05%)	*hla*	37 (38.14%)
*tet*K	56 (72.73%)	*hld*	21 (21.65%)
*bla*Z	71 (88.75%)	*sea*	0 (0.00%)
*mec*A	51 (63.75%)	*seb*	3 (3.09%)
*erm*C	80 (97.56%)	*sed*	2 (2.06%)
*msr*A	15 (18.29%)	*eta*	0 (0.00%)
*aac (6′)-aph (2″)*	5 (62.50%)	*etb*	1 (1.03%)
*van*A	0 (0.00%)	*lukS/F-PVL*	29 (29.90%)
*van*B	0 (0.00%)	*tst*	11 (11.34%)

* The following genes confer resistance to the corresponding antibiotics and were tested in isolates that displayed phenotypic resistance to these antibiotics: *tet*M and *tet*K (tetracycline; *n* = 77), *blaZ* (penicillins; *n* = 80) *ermC, msr*A (erythromycin; *n* = 82), *aac*(6′)-*aph*(2′’) (gentamicin; *n* = 8), *van*A and *van*B (vancomycin; *n* = 67), and *mec*A (methicillin/cefoxitin/β-lactams; *n* = 80). ** The following virulence genes encode the corresponding protein: *hla and hld* (α and δ hemolysins), *eta and etb* (exfoliative toxins), *sea, seb and sed* (staphylococcal enterotoxins), *lukS/F-PVL* (leucocidin), *tst* (toxic-shock syndrome/exotoxin).

## Data Availability

All data are contained within this article and supplementary file.

## Supplementary Materials

The following are available online at https://www.mdpi.com/2076-0817/10/3/317/s1, Table S1: Multidrug resistance profiles of *S. aureus* isolates, Table S2: List of primers used to detect antibiotic resistance and virulence genes.
