# Supplementary material for: Staphylococcus aureus in Intensive Pig Production in South Africa: Antibiotic Resistance, Virulence Determinants, and Clonality
Source: Pathogens. 2021 Mar 8;10(3):317. doi: 10.3390/pathogens10030317 (PMC8000748; doi:10.3390/pathogens10030317)
Supplement: Supplementary file 1 [file pathogens-10-00317-s001.pdf]

Supplementary materials

Staphylococcus aureus in intensive pig production in South Africa: antibiotic resistance, virulence determinants, and clonality

Ncomeka Sineke [1], Jonathan Asante [1], Daniel Gyamfi Amoako [1,2,3,*], Akebe Luther King Abia [1,*], Keith Perrett [4], Linda A. Bester [2] and Sabiha Y. Essack [1]

1 Antimicrobial Research Unit, College of Health Sciences, University of KwaZulu-Natal, Durban 4000, South Africa; nsineje@gmail.com (N.S.); josante33@yahoo.com (J.A.); lutherkinga@yahoo.fr (A.L.K.A.); amoakodg@gmail.com (D.G.A.); essacks@ukzn.ac.za (S.Y.E.)

2 Biomedical Resource Unit, College of Health Sciences, University of KwaZulu-Natal, Durban 4000, South Africa; besterl@ukzn.ac.za (L.A.B.)

3 Centre for Respiratory Diseases and Meningitis, National Institute for Communicable Diseases, Johannesburg 2131, South Africa

4 Epidemiology Section, KwaZulu-Natal Agriculture & Rural Development-Veterinary Service, Pietermaritzburg 3201, South Africa; keith.perrett@kzndard.gov.za (K.P.)

* Correspondence: amoakodg@gmail.com (D.G.A.), Tel.: +27(0)843308957; ORCID: 000-0003-3551-3458; lutherkinga@yahoo.fr (A.L.K.A.), Tel.: +27(0)734403343, ORCID: 000-0002-5194-2810

Table S1: Multidrug resistance profiles of *S. aureus* isolates.

MDR PATTERN	Frequency
LZD-FOX-RIF-ERY-AMK-GENT-TIG-CLI-AMP-PEN-SXT-CIP-MXF-LVX-TET-DOX-NIT-CHL-TEC	1
LZD-FOX-RIF-ERY-CLI-GENT-AMP-PENG-SXT-MXF-TET-DOX-NIT-CHL-VAN-TEC	2
LZD-FOX-RIF-ERY-CLI-GENT-AMP-PEN-SXT-MXF-TET-DOX-NIT-CHL-VAN-TEC	1
LZD-FOX-RIF-ERY-CLI-AMP-PEN-SXT-MXF-TET-DOX-NIT-CHL-VAN-TEC	6
LZD-FOX-RIF-ERY-CLI-AMP-PEN-SXT-CIP-MXF-TET-DOX-NIT-CHL-VAN-TEC	1
LZD-RIF-ERY-CLI-GENT-TIG-AMP-PEN-MFX-DOX-TET-SXT-CHL-NIT-VAN-TEC	1
LZD-FOX-RIF-ERY-CLI-GENT-TIG-AMP-PEN-DOX-TET-SXT-CHL-NIT-VAN-TEC	1
LZD-RIF-ERY-GENT-CLI-AMP-PEN-DOX-TET-SXT-CIP-MXF-LVX-NIT-CHL-VAN-TEC	1
LZD-FOX-RIF-ERY-TIG-CLI-AMP-PEN-DOX-TET-CHL-SXT-NIT-VAN-TEC	1
FOX-RIF-ERY-AMK-GENT-CLI-PEN-DOX-TET-SXT-CIP-MXF-LVX-CHL-VAN	1
LZD-RIF-ERY-CLI-AMP-PEN-SXT-MXF-TET-DOX-NIT-CHL-VAN-TEC	2
LZD-RIF-ERY-CLI-AMP-PEN-SXT-MXF-TET-DOX-NIT-CHL-VAN-TEC	8
LZD-FOX-RIF-ERY-CLI-TIG-TET-DOX-AMP-PEN-SXT-MXF-CHL-VAN-TEC	1
LZD-RIF-ERY-CLI-AMP-PEN-SXT-MXF-TET-DOX-NIT-CHL-VAN-TEC	1
LZD-RIF-ERY-GENT-CLI-AMP-PEN-DOX-TET-CHL-SXT-NIT-VAN-TEC	1
LZD-FOX-RIF-ERY-CLI-AMP-PEN-DOX-TET-SXT-NIT-CHL-TEC	1
LZD-FOX-RIF-ERY-CLI-TIG-AMP-PEN-SXT-TET-DOX-NIT-CHL-TEC	1
LZD-RIF-ERY-CLI-TIG-AMP-PEN-DOX-TET-SXT-MXF-CHL-NIT-VAN-TEC	1
LZD-RIF-ERY-CLI-PEN-SXT-TET-DOX-NIT-CHL-TEC	1
LZD-FOX-RIF-ERY-CLI-TIG-AMP-PEN-SXT-TET-DOX-NIT-VAN-TEC	1
LZD-RIF-ERY-CLI-AMP-PEN-SXT-TET-DOX-NIT-CHL-VAN-TEC	7
LZD-RIF-ERY-CLI-AMP-PEN-SXT-TET-DOX-NIT-CHL-TEC	1
LZD-FOX-RIF-ERY-CLI-GENT-AMP-PEN-SXT-MXF-CIP-LVX-CHL-VAN-TEC	1
LZD-RIF-ERY-CLI-TIG-AMP-PEN-SXT-TET-DOX-NIT-CHL-VAN-TEC	1
LZD-RIF-ERY-CLI-AMP-PEN-SXT-MXF-TET-DOX-VAN-TEC	1
RIF-ERY-CLI-AMP-PEN-SXT-TET-DOX-CHL-TEC	1
LZD-FOX-RIF-ERY-CLI-PEN-MXF-DOX-VAN-TEC	1
LZD-RIF-ERY-CLI-AMP-PEN-TET-DOX-NIT-CHL-VAN-TEC	2
LZD-RIF-ERY-CLI-AMP-PEN-TET-DOX-NIT-CHL-TEC	1
LZD-RIF-ERY-CLI-PEN-SXT-TET-DOX-VAN-TEC	1
LZD-FOX-RIF-ERY-CLI-PEN-NIT-VAN-TEC	1
RIF-ERY-CLI-PEN-DOX-TET-CHL-VAN	1
ERY-CLI-PEN-TET-LVX-VAN	1
ERY-CLI-PEN-SXT-TET-DOX-VAN	1
RIF-ERY-CLI-PEN-TET-DOX-VAN	2
ERY-CLI-AMP-PEN-TET-DOX-CHL-VAN	1
ERY-CLI-AMP-PEN-SXT-TET-DOX-CHL	1
ERY-CLI-AMP-PEN-SXT-TET-DOX-TEC	1
ERY-CLI-PEN-SXT-TET-DOX-VAN	1
ERY-CLI-AMP-SXT-TET-DOX	1
ERY-CLI-PEN-TET-DOX-VAN	2
ERY-CLI-AMP-PEN-SXT-TET-DOX	1

LZD-ERY-CLI-TET-VAN	1
RIF-ERY-PEN-TET-DOX	1
ERY-CLI-DOX-TET-SXT-NIT	1
ERY-CLI-AMP-PEN-SXT-TET-DOX-VAN	1
ERY-CLI-DOX-TET-SXT	1
ERY-CLI-TET-VAN	1
ERY-CLI-DOX-TET-SXT	1
ERY-CLI-AMP-PEN-DOX-TET	1
CLI-PEN-TET-DOX	1
ERY-CLI-TET-VAN	1
ERY-CLI-DOX-TET-VAN	2
AMP-PEN-SXT-VAN	1
ERY-CLI-PEN-SXT	1
ERY-CLI-TET-DOX-VAN	1

Table S2: List of primers used to detect antibiotic resistance and virulence gene.

Gene	Primer/sequence	PCR conditions	PCR size (bp)	Reference
**Resistance**				
mecA	**F**-AACAGGTGAATTATTAGCACTTGTAAG<br>**R**-ATTGCTGTTAATATTTTTTGAGTTGAA	30 s 94 °C, 30 s 55 °C, 1 min 72 °C	174	(Kuntova et al., 2012)
blaZ	**F**-ACTTCAACACCTGCTGCTTT<br>**R**-TGACCACTTTTATCAGCAAC	30 s 94 °C, 30 s 55 °C, 1 min 72 °C	173	(Kuntova et al., 2012)
ermC	**F**-CTTGTTGATCACGATAATTTCC<br>**R**-ATCTTTTAGCAAACCCGTATTC	30 s 94 °C, 30 s 55 °C, 1 min 72 °C	190	(Kuntova et al., 2012)
aac(6′)-aph(2′′)	**F**-TAATCCAAGAGCAATAAGGGC<br>**R**-GCCACACTATCATAACCACTA	30 s 94 °C, 30 s 55 °C, 1 min 72 °C	227	(Kuntova et al., 2012)
msrA	**F**-TCCAATCATAGCACAAAATC<br>**R-**AATTAAATATATTTGGTCGT	30 s 94 °C, 30 s 55 °C, 1 min 72 °C	163	(Kuntova et al., 2012)
tetK	**F**-TCGATAGGAACAGCAGTA<br>**R**-CAGCAGATCCTACTCCTT	30 s 94 °C, 30 s 55 °C, 1 min 72 °C	169	(Kuntova et al., 2012)
tetM	**F-**AGTGGAGCGATTACAGAA<br>**R-**CATATGTCCTGGCGTGTCTA	30 s 94 °C, 30 s 55 °C, 1 min 72	158	(Kuntova et al., 2012)
**Virulence**				
Hla	**F**-CTGATTACTATCCAAGAAATTCGATTG<br>**R**-CTTTCCAGCCTACTTTTTTATCAGT	30 s 95 °C, 45 s 58 °C, 1 min 72 °C	209	(Hoseini Alfatemi et al., 2014)
Hld	**F**AAGAATTTTTATCTTAATTAAGGAAGGAGTG<br>**R**-TTAGTGAATTTGTTCACTGTGTCGA	30 s 95 °C, 45 s 58 °C, 1 min 72 °C	111	(Hoseini Alfatemi et al., 2014)
Eta	**F**-GCAGGTGTTGATTTAGCATT<br>**R**-AGATGTCCCTATTTTTGCTG	30 s 95 °C, 45 s 54 °C, 1 min 72 °C	93	(Hoseini Alfatemi et al., 2014)
Etb	**F-**ACAAGCAAAAGAATACAGCG<br>**R**-GTTTTTGGCTGCTTCTCTTG	30 s 95 °C, 45 s 54 °C, 1 min 72 °C	226	(Hoseini Alfatemi et al., 2014)

Sea	**F-**GGTTATCAATGTGCGGGTGG<br>**R**-CGGCACTTTTTTCTCTTCGG	30 s 94 °C, 30 s 55 °C, 1 min 72 °C	102	(Hoseini Alfatemi et al., 2014)
Seb	**F**-GTATGGTGGTGTAACTGAGC<br>**R**-CCAAATAGTGACGAGTTAGG	30 s 94 °C, 30 s 55 °C, 1 min 72 °C	164	(Hoseini Alfatemi et al., 2014)
Sed	**F-**CCAATAATAGGAGAAAATAAAAG<br>**R**-ATTGGTATTTTTTTTCGTTC	30 s 94 °C, 30 s 55 °C, 1 min 72 °C	278	(Hoseini Alfatemi et al., 2014)
Tst	**F-**ACCCCTGTTCCCTTATCATC<br>**R**-TTTTCAGTATTTGTAACGCC	30 s 94 °C, 30 s 60 °C, 1 min 72 °C	326	(Hoseini Alfatemi et al., 2014)
LukS/F-PV	**F-**<br>ATCATTAGGTAAAATGTCTGGACATGATCCA<br>**R**-GCATCAAGTGTATTGGATAGCAAAAGC	30 s 95 °C, 45 s 60 °C, 1 min 72 °C	443	(Hoseini Alfatemi et al., 2014)
